# Ferrocene-Labelled Electroactive Aptamer-Based Sensors (Aptasensors) for Glycated Haemoglobin

**DOI:** 10.3390/molecules26237077

**Published:** 2021-11-23

**Authors:** Xue-Qing Feng, Yi Ju, Wei-Tao Dou, Qing Li, Zhong-Gan Jin, Xiao-Peng He, Tony D. James, Bang-Ce Ye

**Affiliations:** 1 Laboratory of Biosystem and Microanalysis, State Key Laboratory of Bioreactor Engineering, East China University of Science and Technology, Shanghai 200237, China; fengxueqing@sccl.org.cn (X.-Q.F.); juyi@sccl.org.cn (Y.J.); 2 Shanghai Center for Clinical Laboratory, Shanghai 200237, China; liqing@sccl.org.cn (Q.L.); jinzhonggan@sccl.org.cn (Z.-G.J.); 3 Key Laboratory for Advanced Materials and Joint International Research Laboratory of Precision Chemistry and Molecular Engineering, Feringa Nobel Prize Scientist Joint Research Center, Frontiers Center for Materiobiology and Dynamic Chemistry, School of Chemistry and Molecular Engineering, East China University of Science and Technology, 130 Meilong Rd., Shanghai 200237, China; xphe@ecust.edu.cn; 4 Department of Chemistry, University of Bath, Bath BA2 7AY, UK; 5 School of Chemistry and Chemical Engineering, Henan Normal University, Xinxiang 453007, China

**Keywords:** glycated haemoglobin, aptamer, electrochemical sensor

## Abstract

Glycated haemoglobin (HbA_1c_) is a diagnostic biomarker for type 2 diabetes. Traditional analytical methods for haemoglobin (Hb) detection rely on chromatography, which requires significant instrumentation and is labour-intensive; consequently, miniaturized devices that can rapidly sense HbA_1c_ are urgently required. With this research, we report on an aptamer-based sensor (aptasensor) for the rapid and selective electrochemical detection of HbA_1c_. Aptamers that specifically bind HbA_1c_ and Hb were modified with a sulfhydryl and ferrocene group at the 3′ and 5′-end, respectively. The modified aptamers were coated through sulfhydryl-gold self-assembly onto screen printed electrodes, producing aptasensors with built in electroactivity. When haemoglobin was added to the electrodes, the current intensity of the ferrocene in the sensor system was reduced in a concentration-dependent manner as determined by differential pulse voltammetry. In addition, electrochemical impedance spectroscopy confirmed selective binding of the analytes to the aptamer-coated electrode. This research offers new insight into the development of portable electrochemical sensors for the detection of HbA_1c_

## 1. Introduction

The American Diabetes Association and World Health Organization have reported that blood concentrations of HbA_1c_ over 6.5% are linked with the development of diabetes mellitus [1,2,3]. Accurate detection of HbA_1c_ is of significant clinical value in screening, early diagnosis, treatment, and monitoring of complications associated with diabetes. Currently, a number of analytical methods for HbA_1c_ detection are available for clinical practice, including ion exchange chromatography, gel electrophoresis, turbidity immunoassay and enzymatic methods [4,5,6,7]. However, owing to issues such as the requirement of operational complexity of the instrumentation and cost, miniaturized sensors that can rapidly detect HbA_1c_ are urgently needed. Compared with the existing technology, aptamer-based electrochemical biosensors exhibit many advantages including (1) extended stability, (2) minimal blood for testing is required, and (3) using an aptamer [8,9,10,11] means that cast-type screen printing electrode construction of electrochemical sensors [12,13,14,15] can be used, which is convenient, enables miniaturization, and is low cost. Testing of HbA_1c_ is commonly recommended by several countries for monitoring the development of diabetes. The Chinese Medical Association’s China Guideline for Type 2 Diabetes (2020 edition) [16] designated that laboratory measurements with strict quality control and the standardized test method for determination of glycosylated haemoglobin (HbA_1c_) can be used as a diagnostic criterion for diabetes, where the threshold for the diagnosis of diabetes is 6.5% HbA_1c_ or higher. According to the results of the laboratory quality assessment conducted by the Clinical Laboratory Center of the National Health Commission and the Shanghai Clinical Laboratory Center, standardization of the HbA_1c_ test in China has gradually improved, but there are still large differences amongst different regions and methods [17]. Encouragingly, since the quality of current HbA_1c_ monitoring systems are sub-optimal, excellent development opportunities and prospects exist. At present, the HbA_1c_ measurement instruments used in clinical laboratories in China are mostly imported and are very expensive. Therefore, development of an accurate, rapid, economical, and practical detection method is the goal of this research.

**Scheme 1:**
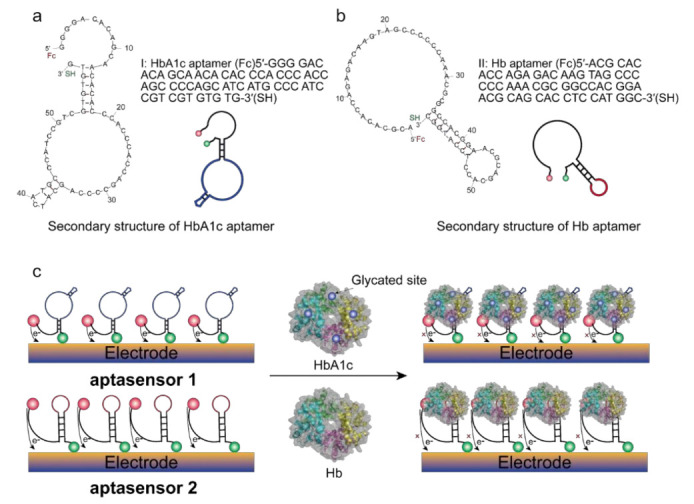
Representative predicted secondary structures of (**a**) Hb aptamer ((Fc)5′-GGG GAC ACA GCA ACA CAC CCA CCC ACC AGC CCC AGC ATC ATG CCC ATC CGT CGT GTG TG-3′(SH)), and (**b**) HbAlc aptamer ((Fc)5′-ACG CAC ACC AGA GAC AAG TAG CCC CCC AAA CGC GGC CAC GGA ACG CAG CAC CTC CAT GGC-3′(SH)) simulated using Mfold software. (**c**) Schematic illustration of the construction of aptasensors **1** and **2** through sulfhydryl-gold self-assembly on screen printed electrodes, and the plausible sensing mechanism of the functionalized aptasensors for haemoglobin (Hb) (with aptasensor (**1**) and glycated haemoglobin (HbA1c) (with aptasensor (**2**); the presence of the proteins increases the distance from the electroactive ferrocene to the electrode surface, thereby impeding the electron transfer process.

Consequently, with this research, we developed a simple, efficient, and low-cost electrochemical biosensor (Scheme 1) [18,19,20,21,22], which uses a low-cost screen-printed electrode as the test electrode. Two aptamer sequences were synthesized according to a previous study [23] with ferrocene and sulfhydryl denoted as Fc and SH, respectively:

I: HbA_1c_ aptamer (Fc)5′-GGG GAC ACA GCA ACA CAC CCA CCC ACC AGC CCC AGC ATC ATG CCC ATC CGT CGT GTG TG-3′(SH) 

II: Hb aptamer (Fc)5′-ACG CAC ACC AGA GAC AAG TAG CCC CCC AAA CGC GGC CAC GGA ACG CAG CAC CTC CAT GGC-3′(SH).

The aptamer system is constructed using a ferrocene as an electrical signal unit linked through the thiol self-assembled coating to a screen-printed gold electrode. After the HbA_1c_ aptamer interacts with a specific protein, the electrical signal of the ferrocene in solution changes. The ratio between HbA_1c_ and non-HbA_1c_ can then be used to predict the occurrence of diabetes mellitus. Upon binding of the aptamer with HbA_1c_, the long distance between ferrocene and electrode hinders the electron transfer, thereby diminishing current intensity [24].

## 2. Results and Discussion

**Figure 1:**
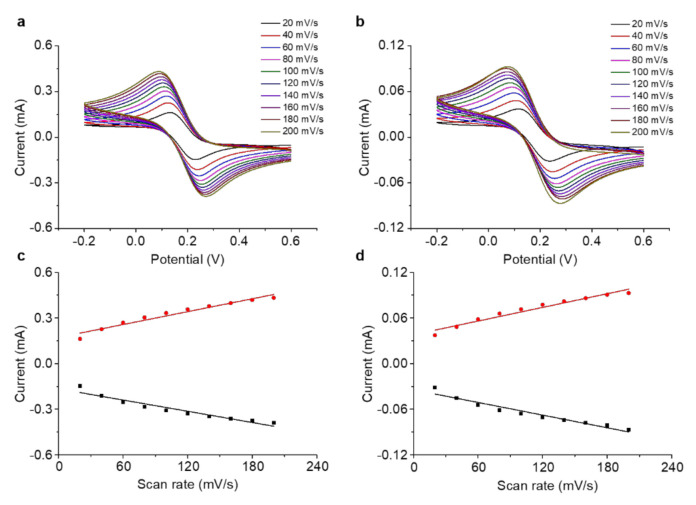
Cyclic voltametric curves of (**a**) aptasensor **1** and (**b**) aptasensor **2** with different scanning rates. Plotting the current intensity of (**c**) aptasensor **1** and (**d**) aptasensor **2** as a function of potential.

In order to evaluate the use of cyclic voltammetry (CV) with the HbA_1c_ and Hb aptamer sensors, the sweep speed was set as 0.02–0.2 V/s and the scanning range used was 0.2–0.6 V. The cyclic voltammograms of the HbA_1c_ aptamer sensor (aptasensor **1**) (Figure 1a) and Hb aptamer sensor (aptasensor **2**) (Figure 1b) indicated that the aptamer sensors exhibited a Faradaic peak of 0.22 V and an oxidation peak of 0.13 V; the two peaks are symmetric with a peak separation of 0.09 V. According to the plot of the scanning speed against the test current, the current of the aptamer sensor in the electrolyte was proportional to the scanning speed. This demonstrated that the chemical behaviour was controlled by interfacial electron transfer. The correlation equations of the oxidation and reduction peak for the HbA_1c_ aptasensor (Figure 1c) were *y* = 10^−4^ + 10^−6^ (HbA_1c_ oxidation peak) (*R*^2^ = 0.948) and *Y* = −10^−4^ − 10^−6^*x* (HbA_1c_ reduction peak) (*R*^2^ = 0.918), respectively. The correlation equations of the oxidation and reduction peak for the Hb aptasensor (Figure 1d) were *Y* = 4 × 10^−5^ + 3 × 10^−7^*x* (Hb oxidation peak) (*R*^2^ = 0.950) and *Y* =−3 × 10^−5^ − 3 × 10^−7^*x* (Hb reduction peak) (*R*^2^ = 0.938), respectively. The amount (Г*) of aptamer **1** and **2** immobilized on the electrode was calculated to be 3.98 × 10^−11^ mol cm^−2^ and 1.89 × 10^−11^mol cm^−2^, respectively, according to the following equation Г* = Q × (nFA)^−1^ [25], where Q is the charge involved in the reaction, n is the number of transferred electrons, F is the Faraday constant and A is the geometric area of the electrode. 

**Figure 2:**
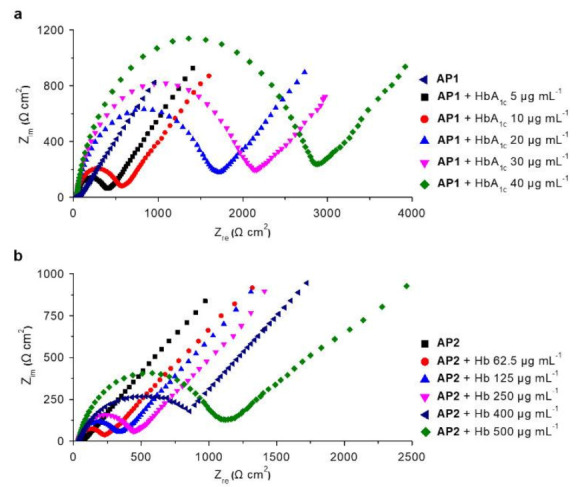
Impedimetric changes of (**a**) aptasensor **1** (**AP1**) with increasing HbA_1c_ and (**b**) aptasensor **2** (**AP2**) with increasing Hb measured by AC impedance spectroscopy.

The impedance of the HbA_1c_ aptamer sensor in HbA_1c_ protein solutions with concentrations of 5.0, 10.0, 20.0, 30.0, and 40 μg/mL and the Hb aptamer sensor in Hb protein solutions with concentrations of 62.5 μg/mL, 125.0 μg/mL, 250.0 μg/mL, 500.0 μg/mL, were evaluated in 5 mmol/L [Fe(CN)_6_]^3−^/[Fe(CN)_6_]^4−^ and 0.1 mol/L KCl electrolyte [20,21,22]. The Nyquist plots of the HbA_1c_ and Hb impedance are shown in Figure 2a,b. With increasing protein concentration, the capacitive reactance arc in the high-frequency region gradually increased. More protein binds at higher polarization resistance, which indicates that both proteins had a certain blocking effect on the current transfer in the test solution, and the magnitude of the capacitive reactance arc reflects the magnitude of the binding protein action on the aptamer sensor.

**Figure 3:**
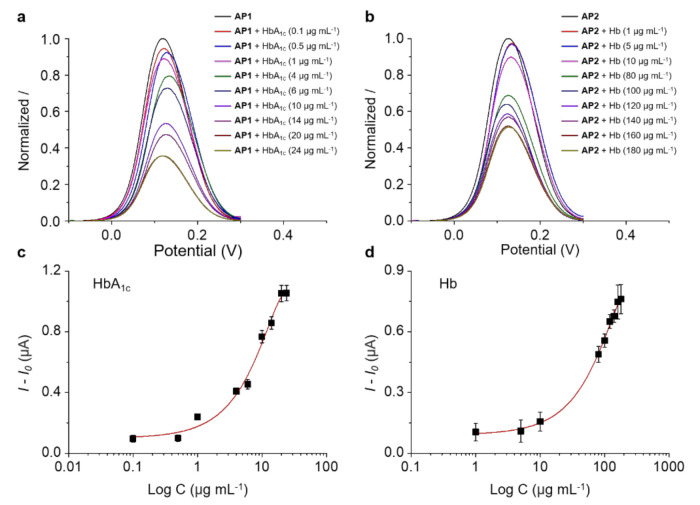
Differential pulse voltametric curves of (**a**) aptasensor **1** (**AP1**) with increasing HbA_1c_ and (**b**) aptasensor **2** (**AP2**). The current intensity (*I*) of an aptasensor in the absence of HbA1c or Hb is normalized to that of the aptasensor in the presence of HbA1c or Hb. (**c**) Plotting the current changes of aptasensor **1** (**AP1**) with increasing HbA_1c_, and (**d**) Plotting the current changes of aptasensor **2** (**AP2**) with increasing Hb, where *I* and *I*_0_ are the current intensity of an aptasensor in the presence and absence of protein analyte, respectively. Error bars mean standard deviation (n = 3).

The differential pulse voltammetry (DPV)-based detection of HbA_1c_ and Hb by the aptamer sensors was achieved according to the protocol shown in the experimental section. The changes of the peak currents after binding of the corresponding proteins to the HbA_1c_ and Hb aptamer sensors were determined by the DPV method (Figure 3a,b, respectively). The aptamer sensor response without protein addition was used as a control to quantitatively detect the corresponding protein. The protein concentration was used as the *X* axis and the peak current was the *Y* axis. The fitting curves of HbA_1c_ and Hb are shown in Figure 3c,d, respectively. The fitting equations and correlation coefficients were as follows: *Y* = 10^−6^*x*/(11.740 + *x*) (*R*^2^ = 0.969) and *Y* = 10^−6^*x*/(95.916 + *x*) (*R*^2^ = 0.968). The dissociation constant (*K*_d_) of HbA_1c_ was 11.7 ± 3.3 μg/mL, and that of Hb was 95.9 ± 40.0 μg/mL as calculated via the equation Y = *B*_max_X/(*K*_d_ + X), where Y is the current intensity of the aptasensor in the presence of a protein, X is the concentration of the protein, and *B*_max_ and *K*_d_ are the slope and intercept of the equation, respectively. Data were analyzed using Origin 8.5 (OriginLab Corporation). The linear range of the aptasensors were determined to be 0.1–14 μg/mL (*R*^2^ = 0.97) and 1–140 μg/mL (*R*^2^ = 0.95) for HbA_1c_ and Hb, respectively. The limit of detection of the aptasensors were calculated to be 84 pg/mL and 237 pg/mL for HbA_1c_ and Hb, respectively (3 σ/k, where σ is the standard deviation of the instrument and k is the slope of the plots shown in Figure 3c,d.

**Figure 4:**
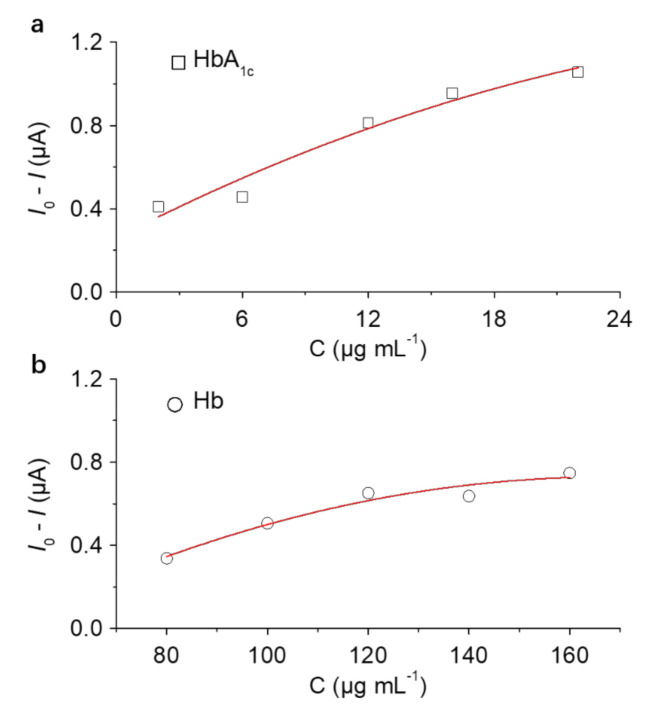
(**a**) Plotting the current changes of aptasensor **1** with increasing HbA_1c_, and (**b**) plotting the current changes of aptasensor **2** with increasing Hb, where *I* and *I*_0_ are the current intensity of an aptasensor in the presence and absence of protein analyte, respectively.

According to the HbA_1c_ and Hb protein concentrations in Table 1, the DPV method was performed according to the established detection method. The protein concentration was used as the *X* axis and the peak current was the *Y* axis (Figure 4a,b); data for a broader protein concentration range are given in Table S1 and Figure S5. The second-order fitting equations for HbA_1c_ and Hb were y = −7 × 10^−10^x^2^ + 5 × 10^−8^x + 3 × 10^−7^ (*R*^2^ = 0.960) and y = −5 × 10^−11^x^2^ + 2 × 10^−8^X −7 × 10^−7^ (*R*^2^ = 0.952) [26], respectively. The results indicated that the HbA_1c_ concentration was in the range 2.0–22.0 μg/mL and the Hb concentration was in the range 80.0–160 μg/mL. The clinical detection range of HbA_1c_ protein/Hb protein was calculated to be in the range of 4.4%–14.7% (25.0–137.5 mmol/mol). The results suggest that the signal change of both aptasensors correlates well to the protein concentration ranges typically detected in the blood, which could be potentially applicable for clinical application. 

Abnormal HbA_1c_ levels are a common pathophysiological basis for type 2 diabetes mellitus (T2DM) and pancreatic cancer, and it is observed long before the clinical symptoms of these diseases appear. Existing methods for the detection and evaluation of diabetes include microcolumn ion-exchange chromatography, affinity chromatography, high-performance liquid chromatography (HPLC), immunofluorescence, ion capture, and electrophoresis. The high-pressure liquid-phase method is considered to be the gold standard for HbA_1c_ detection. At present, clinical laboratories typically use liquid chromatography detection methods for HbA_1c_ detection. However, the dependence on large machines reduces patient compliance, and affects the effective management of diabetes. To date the use of point-of-care (POC) instruments for detection of HbA_1c_ in clinical laboratories has not been investigated. Currently, HbA_1c_ analysers have only been used in clinical trials for POC, so the creation of sensitive micro-HbA_1c_ biosensors is still a work in progress.

Accurate POC devices for HbA_1c_ evaluation are essential, but their development is constrained by the cost of the testing systems. Because T2DM is a progressive disease, early screening of the blood glucose status is an effective method for the treatment and management of early-stage diabetes, and it is very important to reduce the number of missed diabetes diagnoses. The electrochemical biosensor method established in this study for the detection of HbA_1c_ [27,28] is simple to operate and inexpensive, and as such is suitable for general clinical use. The linear relationship of this method is over a range from 4.4% to 14.7% NGSP units and meets the requirements of clinical analysis. DPV tests indicated that the peak currents of both aptamer sensors gradually decreased with increasing protein concentrations. This was because the aptamer bound to the protein and formed a protein coating on the surface of the sensor to block electron transfer of the Fc group. From the Nyquist plots obtained from the impedance experiments, regardless of the presence or absence of the protein (blank), the shapes of the impedance spectra were similar, which was a flattened semicircular capacitive reactance arc with an inclined upward line (Warburg impedance). This “repressed” semicircular capacitive reactance arc is caused by frequency diffusion, which in turn is related to the interface uniformity, defects, and protein adsorption on the electrode surface. The Warburg diffusion line at the end of the spectrum is caused by diffusion. In addition, the size of the capacitive reactance arc reflects the size of the polarization resistance of the system. The polarization resistance is higher for a larger capacitive reactance arc, which means that the corresponding binding current density is smaller (that is, more protein binding).

## 3. Materials and Methods

### 3.1. General

All of the chemicals and reagents used were of analytical grade. Sodium dihydrogen phosphate monohydrate (NaH_2_P_3_O_4_·H_2_O), disodium hydrogen phosphate (Na_2_HP_3_O_4_·H_2_O), magnesium chloride (MgCl_2_), tris (hydroxymethyl) aminoethane (Tris), sodium chloride (NaCl), hydrochloric acid (HCl), sodium hydroxide (NaOH), sulfuric acid (H_2_SO_4_), potassium chloride (KCl), ethanol (CH_3_CH_2_OH), mercaptoethanol (MEH), and potassium ferricyanide (K_3_[Fe(CN)_6_]·3H_2_O and K_4_[Fe(CN)_6_]·3H_2_O) were purchased from Sigma-Aldrich. The disulfide bond reducing agent tris(2-formyl ethyl) phosphine hydrochloride (TCEP) was obtained from Thermo Fisher, Inc. HbA_1c_ aptamer (Figures S1 and S2) and Hb aptamer (Figures S3 and S4) with a purity of 98% were synthesized by Sangon Bioengineering (Shanghai) Co., Ltd. HbA_1c_ protein and Hb protein were purchased from Prospec Corporation. All experiments used ultrapure water (resistivity 18.2 MΩ/cm).

### 3.2. Apparatus

A CHI660E electrochemical workstation (Shanghai CH Instruments Ins., China) and screen-printed electrodes (220AT, Dropsense, Swiss) were used. The screen-printed electrodes consist of a three-electrode system: gold (Au, inner diameter = 4 mm) or aptamer-modified gold as the working electrode, gold as the counter electrode, and a silver electrode used as the reference electrode.

### 3.3. Preparation of Aptamer Biosensor

Electrodes were immersed in a 0.05 mol/L NaOH solution and scanned by CV until the signal is stable with a scanning range of −0.3V–1.3 V and scanning speed of 0.1 V/s. Electrodes were then treated under ultrasound in 100% ethanol and ultrapure water for 3 min. Subsequently, the electrodes were rinsed under ultrasound sequentially in 0.5 mmol/L H_2_SO_4_, 0.1 mmol/L KCl, and ultrapure water, followed by drying with N_2_ prior to use. HbA1c or Hb aptamer was diluted to a final concentration of 5 μmol/L with TM solution (5 mmol/L MgCl and 10 mmol/L Tris-HCl), to which was added a tris(2-formyl ethyl)phosphine hydrochloride solution (TCEP, concentration: 3 mmol/L) to reduce disulfide bonds. Then, the mixture was annealed at 37 °C for 20 min. Next, 3 μL of an aptamer solution was dropped onto the prepared electrodes, which were then incubated at room temperature for 16 h, rinsed three times with phosphate-buffered saline (PBS, 0.1 mol/L, pH 6.5, 1 mM MgCl_2_), and then dried with N_2_ prior to use.

### 3.4. Cyclic Voltammetry (CV)

CV was performed with a computer-controlled CHI760E electrochemical workstation (Chenhua Co., Ltd., Shanghai, China) between 0.2 and 0.6 V (vs. Ag/AgCl) at scan rates ranging from 0 to 200 mV/s. The electrolyte (5 mmol/L K_3_[Fe(CN)_6_], 5 mmol/L K_4_[Fe(CN)_6_]·3H_2_O/0.1 mol/L KCl) was degassed with N_2_ for 20 min before measurements. The screen-printed electrodes (SPEs) were pre-treated in NaOH (0.05 mol/L), H_2_SO_4_ (0.5 mmol/L), H_2_SO_4_ (0.5 mmol/L containing 0.1 mmol/L KCl) solution, and H_2_SO_4_ (0.05 mmol/L) by applying an anodic potential (scanning range −0.2 to 0.6 V, scanning speed 0.1 V/s (vs. Ag/AgCl) for 2 s), and they were then washed with ultrapure water and blown dry with N_2_ for use. The circular area (4 mm in diameter) was used as the working electrode, the reference electrode was printed with gold paste, and the auxiliary electrode was printed with AgCl as silver paste. For functionalization of the SPEs, a drop of the aptamer solution (5 μL) was dropped onto the working electrode and then dried under vacuum. The functionalized SPEs were then immersed in the electrolyte to record the cyclic voltammograms.

### 3.5. Differential Pulse Voltammetry (DPV)

DPV was performed with an amplitude of 0.5 V, a pulse width of 0.05 s, a standing time of 2 s, and a scanning range from −0.1 to 0.3 V in TM (5 mmol/L MgCl_2_, 10 mmol/L Tris-HCl, pH 6.5). For detection of the analytes, drops (5 μL) of the analyte solution (dissolved in phosphate-buffered saline or Tris-HCl) with different concentrations were dropped onto the aptamer-functionalized working electrode area of the SPE and incubated for 90 min. The electrodes were then rinsed with buffer solution three times, dried at room temperature, and immersed in degassed buffer for measurement.

Electrochemical impedance spectroscopy (EIS) [29,30,31,32]. EIS was performed with a CHI760E electrochemical workstation in the presence of an [Fe(CN)_6_]^3−^/[Fe(CN)_6_]^4−^ (5 mmol/L) redox couple in 0.1 M KCl solution over a frequency range from 10 mHz to 100,000 Hz (perturbation signal 5 mV). All of the collected data were fitted using ZSimpWin software. The aptamer solution (5 μL) was first dropped onto the working electrode area of the SPE, dried under vacuum, and then the Nyquist plots were recorded. For detection of the sugar–lectin interactions, a drop of protein solution (Tris-HCl, 5 μL) was dropped onto the aptamer-functionalized electrode [33,34,35,36], dried under vacuum, and then the Nyquist plots were recorded.

### 3.6. Application of The Aptamer Sensors for Clinical Detection

In accordance with the requirements for clinical testing, different concentrations of protein samples with protein molecular weight of 63,000 were tested using the unit conversion formula IFCC (mmol/mol) = 10.93 × NGSP (National Glycohemoglobin Standardization Program) (%) −23.5 by DPV in 10 mmol/L Tris-HCl solution. Each sample was tested three times. The mean of the peak current test results were calculated using the protein concentration as the *X* axis and the peak current as the *Y* axis. Analysis by second-order linear fitting gave the relevant equations, correlation coefficient (*r*), and detection range.

### 3.7. Statistical Analysis

Microsoft Excel software was used to analyze the test data for evaluation of clinical detection application of the adapter sensors. Origin 8.5 software was used to analyze the alternating current (AC) impedance test data of the protein on the adapter sensor, method for detecting HbA_1c_ and Hb by the adapter sensor, and the test data of the influence of the scanning speed on the signal of the adapter sensor.

## 4. Conclusions

The reported method has the advantages of small sample size, convenient operation, miniaturization, and low cost. We are currently working to expand our system to monitor both healthy and diseased populations in China in order to provide reference data for detection of HbA_1c_ using our POC system.

## Figures and Tables

**Table 1:** Concentration of HbA_1c_ and Hb, and clinically relevant detection range of HbA_1c_ and Hb.

Species	Units	Con.1	Con.2	Con.3	Con.4	Con.5
HbA_1c_	μg/mL	2	6	12	16	22
Hb	μg/mL	80	100	120	140	160
HbA_1c_/Hb	mmol/mol	25	60	100	114	137.5
NGSP	%	4.4	7.6	11.3	12.6	14.7

## Data Availability

Not applicable.

## Supplementary Materials

The following are available online, Figure S1: Mass spectrum of HbA_1c_ aptamer (I), Figure S2: HPLC of HbA_1c_ aptamer (I), Figure S3: Mass spectrum of Hb aptamer (II), Figure S4: HPLC of Hb aptamer (II), Table S1: Concentration used for HbA1c and Hb, Figure S5: Plotting the current changes of the aptasenors as a function of protein concentration.
