# Supplementary material for: Ferrocene-Labelled Electroactive Aptamer-Based Sensors (Aptasensors) for Glycated Haemoglobin
Source: Molecules. 2021 Nov 23;26(23):7077. doi: 10.3390/molecules26237077 (PMC8659020; doi:10.3390/molecules26237077)
Supplement: Supplementary file 1 [file molecules-26-07077-s001.zip › molecules-1444997-supplementary.pdf]

molecules

Supplementary Materials

Ferrocene-Labelled Electroactive Aptamer-Based Sensors (Aptasensors) for Glycated Haemoglobin

Xue-Qing Feng [1,2,†], Yi Ju [1,2,†], Wei-Tao Dou [3,*], Qing Li [2], Zhong-Gan Jin [2], Xiao-Peng He [3], Tony D. James [4,5*] and Bang-Ce Ye [1,*]

[1] Laboratory of Biosystem and Microanalysis, State Key Laboratory of Bioreactor Engineering, East China University of Science and Technology, Shanghai 200237, China; fengxueqing@sccl.org.cn (X.-Q.F.); juyi@sccl.org.cn (Y.J.)
[2] Shanghai Center for Clinical Laboratory, Shanghai 200237, China; liqing@sccl.org.cn (Q.L.); jinzhonggan@sccl.org.cn (Z.-G.J.)
[3] Key Laboratory for Advanced Materials and Joint International Research Laboratory of Precision Chemistry and Molecular Engineering, Feringa Nobel Prize Scientist Joint Research Center, Frontiers Center for Materiobiology and Dynamic Chemistry, School of Chemistry and Molecular Engineering, East China University of Science and Technology, 130 Meilong Rd., Shanghai 200237, China; xphe@ecust.edu.cn (X.-P.H.)
[4] Department of Chemistry, University of Bath, Bath, BA2 7AY, UK
[5] School of Chemistry and Chemical Engineering, Henan Normal University, Xinxiang 453007, China
* Correspondence: douweitao123@163.com (W.-T.D.); chstdj@bath.ac.uk (T.D.J.); bcye@ecust.edu.cn (B.-C.Y.)
† Equal contribution.

Table of Contents	Page
Figure S1: Mass spectrum of HbA_{1c} aptamer (I).	2
Figure S2: HPLC of HbA_{1c} aptamer (I).	3
Figure S3: Mass spectrum of Hb aptamer (II).	4
Figure S4: HPLC of Hb aptamer (II).	5
Table S1: Concentration used of HbA1c and Hb.	6
Figure S5: Plotting the current changes.	7

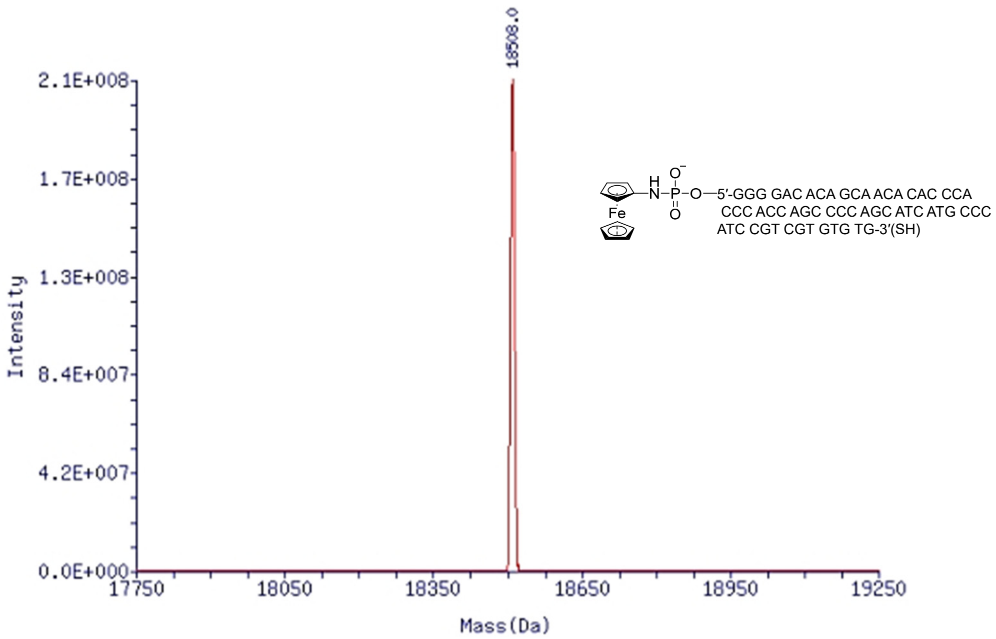

Figure S1. Mass spectrum of HbA_{1c} aptamer (I). (Fc)5′-GGG GAC ACA GCA ACA CAC CCA CCC ACC AGC CCC AGC ATC ATG CCC ATC CGT CGT GTG TG-3′(SH), [M]: calcd. 18506.1, found 18508.0.

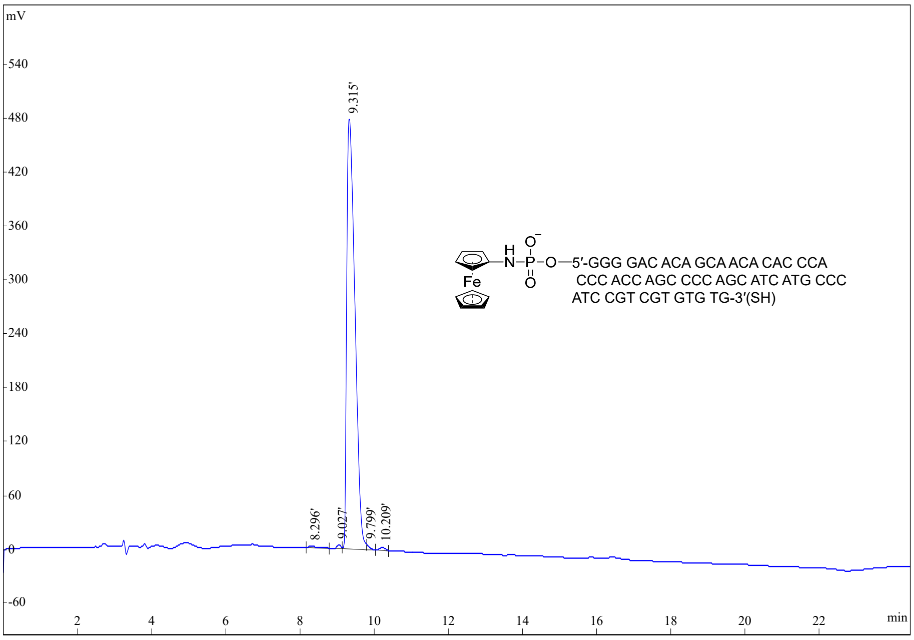

Figure S2. HPLC of HbA_{1c} aptamer (I). t_R = 9.315 min over 22 min with 1.0 mL min^{-1} mobile phase (10% water and 90% methanol), purity 98.1%.

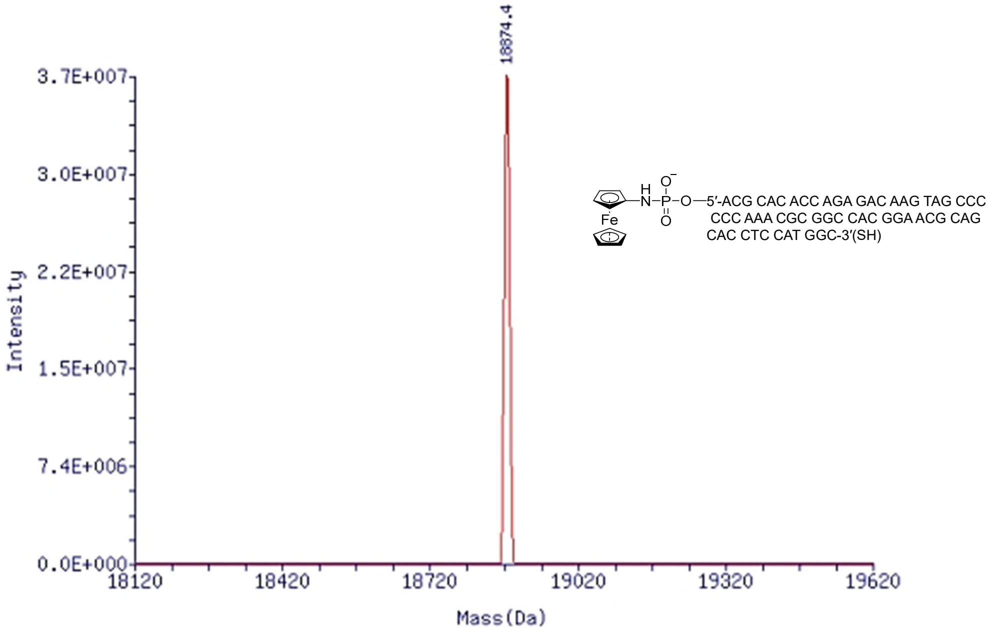

Figure S3. Mass spectrum of Hb aptamer (II). (Fc)5′-ACG CAC ACC AGA GAC AAG TAG CCC CCC AAA CGC GGC CAC GGA ACG CAG CAC CTC CAT GGC-3′(SH), [M]: calcd. 18870.3, found 18874.4.

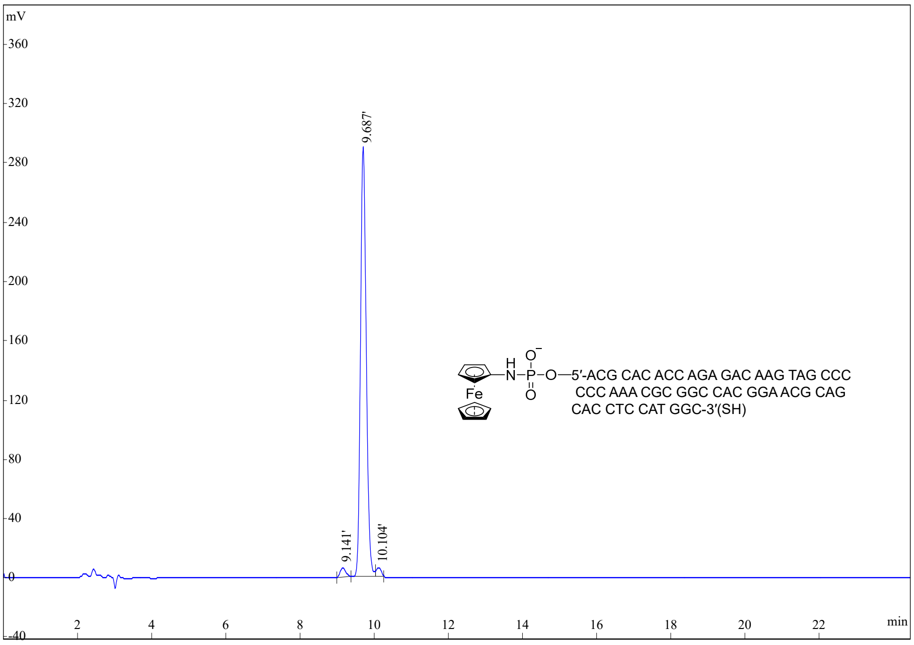

Figure S4. HPLC of Hb aptamer (II). t_R = 9.687 min over 22 min at 1.0 mL min^{-1} mobile phase (10% water and 90% methanol), purity 98.4%.

Table S1. Concentrations of HbA1c and Hb using aptasensor **1** and **2**, respectively.

**Species**	**Units**	**Con. 1**	**Con. 2**	**Con. 3**	**Con. 4**	**Con. 5**	**Con. 6**	**Con. 7**
HbA_{1c}	μg/mL	1	2	6	12	16	22	30
Hb	μg/mL	80	100	120	140	160	180	200

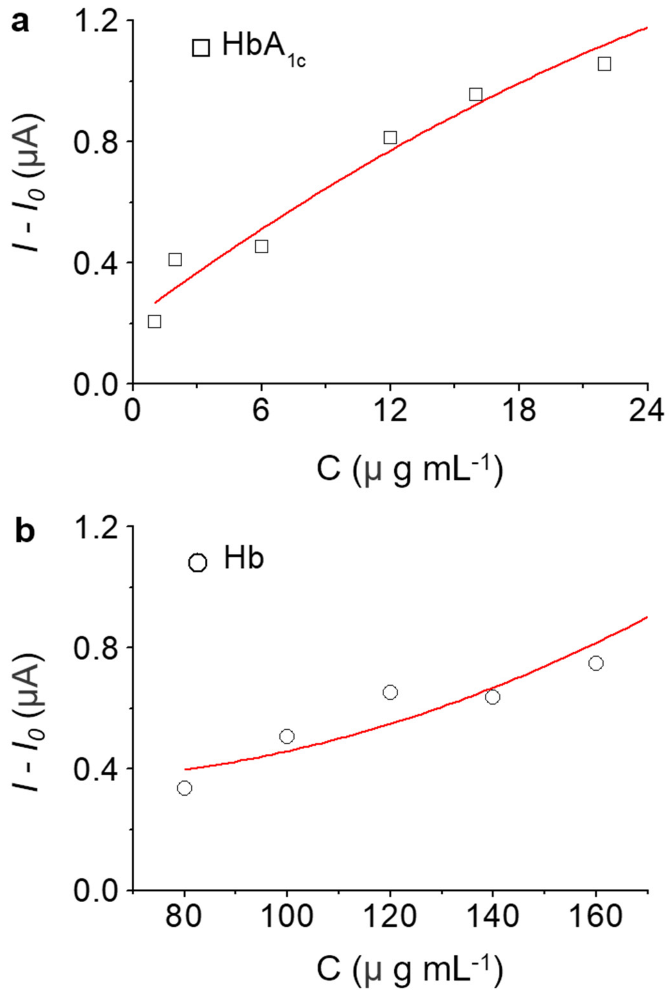

Figure S5. (**a**) Plot for the current changes of aptasensor **1** with increasing HbA1c (1，2，6，12，16，22 and 30 μg mL^{-1}), and (**b**) plot of the current changes of aptasensor **2** with increasing Hb (80，100，120，140，160，180 and 200 μg mL^{-1}), where I and I_0 are the current intensity of the aptasensor in the presence and absence of protein analyte, respectively.
